# Atypical Oral Mucosal Lesions in Syphilis: A Case Report Highlighting the Diagnostic and Therapeutic Aspects of the "Great Imitator"

**DOI:** 10.7759/cureus.75370

**Published:** 2024-12-09

**Authors:** Dorota Szydłowska, Aleksandra Morajko, Karolina Zarańska, Aleksandra Kapuśniak, Grażyna Wąsik

**Affiliations:** 1 Faculty of Medicine, University of Opole, Opole, POL; 2 General and Oncological Dermatology Ward with a Day Care Unit, Provincial Hospital, Opole, POL

**Keywords:** early syphilis, primary symptom, serology, sexually transmitted infections, treponema pallidum

## Abstract

The diagnostic process and discrimination of mucosal lesions present a formidable challenge for numerous clinicians, primarily attributable to the common overlap of clinical manifestations observed across various categories, including infectious, autoimmune, connective tissue, and systemic vascular inflammatory diseases. In cases of mucosal lesions, syphilis presents distinctive characteristics that can help clinicians differentiate it from other conditions. The most common manifestation of primary syphilis is mostly a painless, firm, indurated ulcer known as a chancre, which typically appears at the site of inoculation, with enlargement of regional lymph nodes. Sometimes, its painless course may be misleading. The objective of our study is to raise awareness of venereal diseases in cases of atypical oral mucosal lesions that do not respond to local treatment.

A 32-year-old man was referred to the department of dermatology due to an enlarging, painful inflammatory lesion with central breakdown, covered with honey-yellow crusts on the lower lip. A positive history of herpes labialis was noted. The patient denied risky sexual behaviors or new partners in the last three months. Submandibular and left cervical lymph nodes were found enlarged in ultrasound examination, and laboratory tests showed elevated inflammatory parameters. Empirical antibiotic therapy with amoxicillin-clavulanate in conjunction with oral acyclovir did not lead to clinical improvement. The inadequate therapeutic response prompted the pursuit of further diagnostic investigations, including serological tests encompassing both treponemal and nontreponemal tests. Co-infections with human immunodeficiency virus (HIV) and hepatotropic viruses were excluded. The serological testing resulted positive, and after receiving the diagnosis, the patient admitted to engaging in high-risk sexual behaviors. The treatment with benzathine penicillin was successful.

In the differential diagnosis of ulcers located on mucous membranes or vermilion border, the primary symptom should always be considered. Early serological testing remains the gold standard for diagnosis. Appropriate treatment with benzathine penicillin leads to satisfactory effects.

## Introduction

Syphilis is one of the most frequently reported sexually transmitted diseases in Poland, with a steadily increasing number of cases [1]. The etiological agent is the spirochete *Treponema pallidum*. Infection most commonly occurs during sexual contact. The disease presents with a highly variable clinical picture, hence often referred to as the "great imitator." The primary symptom, characteristic for early syphilis, appears at the site where the spirochetes inoculate the body. There are cases where, due to an atypical clinical course, the final diagnosis is delayed. Mucosal lesions, similar to those observed in other prevalent infections such as herpes simplex virus or chancroid, can also be seen in syphilis. Due to the overlap in clinical manifestations, differential diagnosis is essential, requiring a high degree of clinical suspicion to accurately identify syphilis [2].

## Case presentation

**Figure 1:**
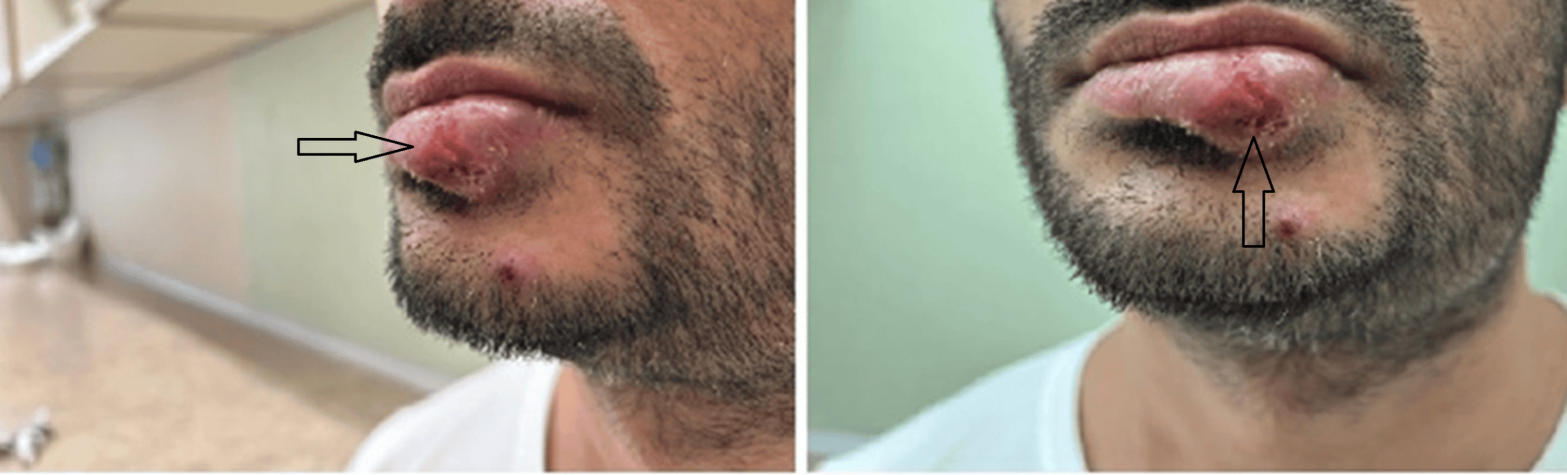
Challenging-to-diagnose, atypical, enlarging, painful inflammatory lesion with central breakdown and honey-yellow crusts on the left side of the lower lip, observed in physical examination

A 32-year-old man was referred to the department of dermatology due to an enlarging, painful inflammatory lesion with central breakdown, covered with honey-yellow crusts on the left side of the lower lip (Figure 1). The skin lesions were accompanied by a rise in body temperature to 37.5°C, excessive sweating, and chills. A positive history of herpes labialis at the place of the lesion was noted.

**Figure 2:**
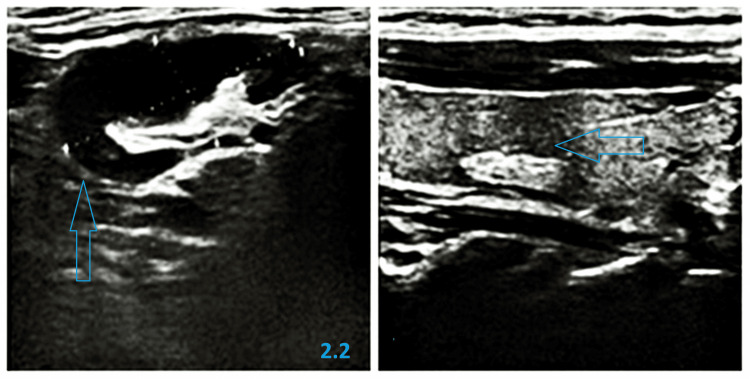
Ultrasound examination of enlarged left cervical lymph nodes 2.2 revealed thickened, hypoechoic cortex up to 36 mm in diameter Both sides: enlarged left cervical lymph nodes with thickened, hypoechoic cortex Left side (2.2): a lymph node with a diameter of 36 mm was observed

The patient denied risky sexual contacts and new partners in the last three months. Additionally, numerous small erosions of the oral mucous membranes and local lymphadenopathy in the submandibular and left cervical region were noted during physical examination. Laboratory tests showed elevated inflammatory parameters: C-reactive protein (CRP) 72 mg/l and leukocytosis in peripheral blood. Ultrasound examination of peripheral lymph nodes revealed numerous enlarged left cervical lymph nodes, up to 36 mm in size, with thickened, hypoechoic cortex and signs of increased vascularity (Figure 2).

Swab culture from the skin lesion yielded only a common skin commensal, *Staphylococcus aureus*, but previous empirical antibiotic therapy did not lead to clinical improvement. Due to the positive history of herpes labialis and secondary lichenification, oral acyclovir therapy in conjunction with fusidic acid applied topically was initiated. The lack of satisfactory therapeutic effect prompted further diagnostic testing, including treponemal and nontreponemal tests. Syphilis was suspected as a diagnosis of exclusion. Co-infection with human immunodeficiency virus (HIV) and hepatotropic viruses was ruled out. A final diagnosis of the herpetiform manifestation of primary syphilis was established based on the following findings: venereal diseases research laboratory (VDRL) 1:32 and *Treponema pallidum* hemagglutination assay (TPHA) 1:640. After hearing the diagnosis, the patient, despite previous denials, admitted to having risky sexual contacts in the last three months, and he was advised of the importance of informing their sexual partner(s) to undergo medical evaluation. Benzathine penicillin G was administered intramuscularly at a dose of 2.4 million units. Serological follow-up tests at three and six months indicated a good response to treatment, with VDRL levels dropping to <1:1 and TPHA levels at 1:320 after three months. VDRL remaining <1:1 and TPHA reducing to 1:80 were observed after six months. The patient is currently in full physical and mental health, and the next follow-up visit is scheduled for six months from now.

## Discussion

Sexually transmitted infections (STIs), including syphilis, represent a significant global public health issue. In Poland, syphilis is one of the most commonly reported STIs, with men being affected approximately nine times more frequently than women. Recent epidemiological data show an upward trend in syphilis cases, despite a temporary decline in 2020. In 2021, 25,270 confirmed cases of syphilis were reported in 28 European Union (EU)/European Economic Area (EEA) countries, marking an increase compared to previous years, particularly after a decline in 2020 for the first time in eight years [3]. Syphilis remains a major concern in both developed and developing countries, where it is especially prevalent among populations with limited access to healthcare [4].

The highest risk of syphilis infection is observed in individuals with HIV, particularly men who have sex with men (MSM). Identifying high-risk groups for regular surveillance is essential to controlling the spread of the disease [5].

Syphilis progresses through distinct stages: primary, secondary, latent, and tertiary. The primary stage is characterized by the appearance of a solitary, painless nodule that evolves into an ulcer with a clean, shiny base and serous exudate at the site of *Treponema pallidum* inoculation and appears between three weeks and three months after exposure. The location of this primary lesion is determined by the nature of sexual contact, and atypical primary lesions are becoming increasingly common due to changing sexual behaviors. Direct contact with infectious mucosal lesions during oral, vaginal, or anal unprotected sexual contact with an infected partner, especially when one or both individuals are unaware of the infection, contributes to the lesions located on the mucous membranes of the mouth, genitalia, or anus as common sites of the inoculation [6].

Sometimes, the presentations of primary lesion may be unusual with herpetiform, giant, gangrenous, or erosive lesions, as well as syphilitic balanitis or vulvar or vaginal inflammation [7,8].

The primary lesion often resolves spontaneously within 2-6 weeks without scarring. However, untreated cases can progress to tertiary syphilis in approximately 30% of cases, which can lead to severe complications such as gummatous syphilis, cardiovascular syphilis, bone and joint involvement, and symptomatic neurosyphilis [9].

In the differential diagnosis of primary syphilis, other conditions such as chancroid, herpes simplex virus infections, squamous cell carcinoma (SCC), and nonspecific ulcers must be considered [10].

Serological testing remains the gold standard for diagnosing syphilis and monitoring treatment efficacy. In the early stages of infection, treponemal tests such as TPHA, *Treponema pallidum* particle agglutination (TPPA), and fluorescent treponemal antibody absorption (FTA-ABS) detect antibodies against *Treponema pallidum *and typically become positive in the third to fourth week of infection. These tests cannot be used to assess treatment efficacy or to differentiate between active and past infections since they may remain positive for life. Nontreponemal tests (e.g., VDRL, rapid plasma reagin (RPR)) detect antibodies directed against cardiolipin, a substance released by damaged cells. These tests are more useful for monitoring disease activity, as their titers decrease with successful treatment [11].

In the primary stage, serological tests may not be immediately positive due to low antibody levels, so a clinical diagnosis is essential. If there is a high suspicion of syphilis based on clinical presentation, additional diagnostic methods such as darkfield microscopy or polymerase chain reaction (PCR) testing can be used to detect *Treponema pallidum* directly, especially in atypical cases [12].

Early detection, appropriate treatment, and patient education are essential to control syphilis. While intramuscular benzathine penicillin G is the first-line treatment for all forms of syphilis, excluding central nervous system syphilis, penicillin remains effective, and *Treponema pallidum* has not shown resistance to it. Alternative therapies are available for those with penicillin allergies, including doxycycline and ceftriaxone [13,14]. Patients should also be educated on the risk of reinfection, as successful treatment does not provide immunity against future infections.

## Conclusions

The purpose of our case description is to pay attention to the need for special venereological vigilance in every case of atypical and unresponsive mucosal lesions. In the differential diagnosis of ulcers located on mucous membranes or vermilion border, the primary symptom of syphilis should always be considered, especially nowadays, when an increasing number of syphilis cases are observed. Serological tests constitute the gold standard in diagnostic evaluation. Benzathine penicillin G, given 2.4 million units intramuscularly, is an effective and safe method of treating syphilis. Early detection and prompt treatment are critical, as untreated syphilis can lead to severe complications affecting multiple organs. Timely diagnosis and intervention are essential to prevent long-term health consequences and to ensure the best patient outcomes.
